# Management of atrioesophageal fistula after catheter ablation for atrial fibrillation: Layered closure with interposition and esophageal stenting

**DOI:** 10.1016/j.xjtc.2024.08.023

**Published:** 2024-09-10

**Authors:** Jenna Aziz, Nahush A. Mokadam, Peter Kneuertz, Bryan A. Whitson

**Affiliations:** a Integrated Cardiothoracic Surgery Residency Program, The Ohio State Wexner Medical Center, Columbus, Ohio; b Division of Cardiac Surgery, Department of Surgery, The Ohio State Wexner Medical Center, Columbus, Ohio; c Division of Thoracic Surgery, Department of Surgery, The Ohio State Wexner Medical Center, Columbus, Ohio

**Figure:**
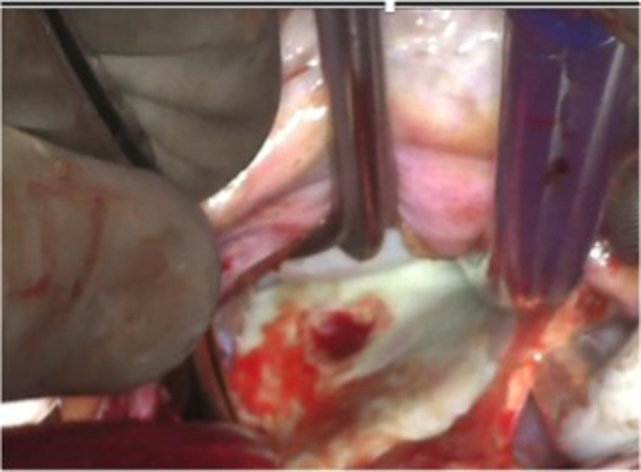
Atrioesophageal fistula: Fistulous tract from within left atrium.

**Central Message**

Atrialesophageal fistula is a rare complication of radiofrequency ablative treatment for atrial fibrillation. This case report describes a successful operative approach for atrioesophageal fistula.

## Case Report

**Figure 1:**
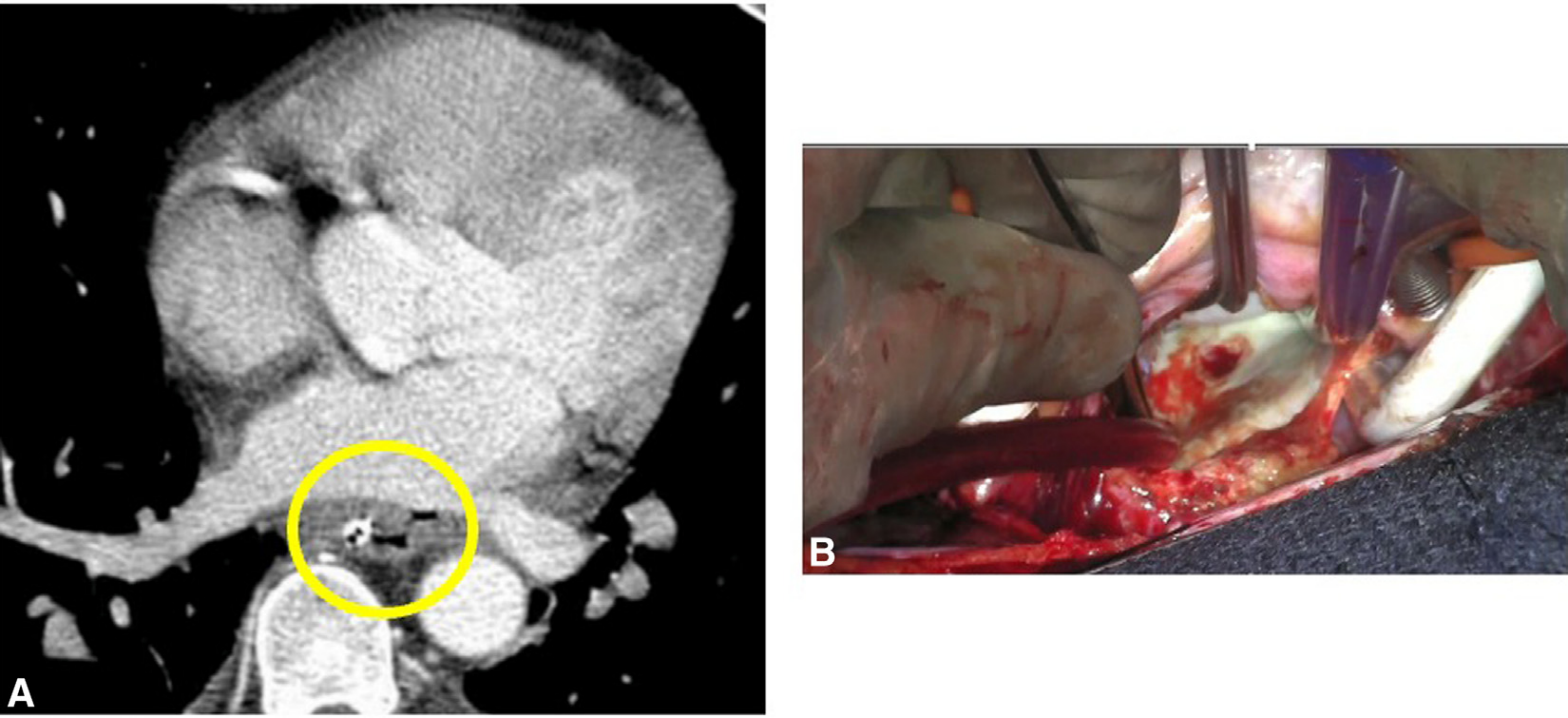
A, Computed tomography scan with air in mediastinum. B, Fistula tract from left atrium.

A 59-year-old man with a history of paroxysmal atrial fibrillation (on apixaban) with prior radio frequency catheter ablation (RFCA) in 2005. Thirty-four days after second RFCA procedure, he presented to an outside hospital with fevers, chills, and diarrhea. He was febrile, at 103 °F; hypotensive; and stabilized with fluids and vasopressors. Mediastinal air was missed on outside chest computed tomography (CT). Blood cultures were positive for *Streptococcus anginosus*. An episode of coffee ground emesis occurred on post-RFCA day 35 with transient upper and lower extremity weakness. Lacunar and basilar infarcts were seen on head magnetic resonance imaging. A reassessment of the chest CT noted air between esophagus and left atrium (LA) (Figure 1, *A*) and he was transferred to our center for management of an atrioesophageal fistula (AEF).

## Surgical Approach

**Figure 2:**
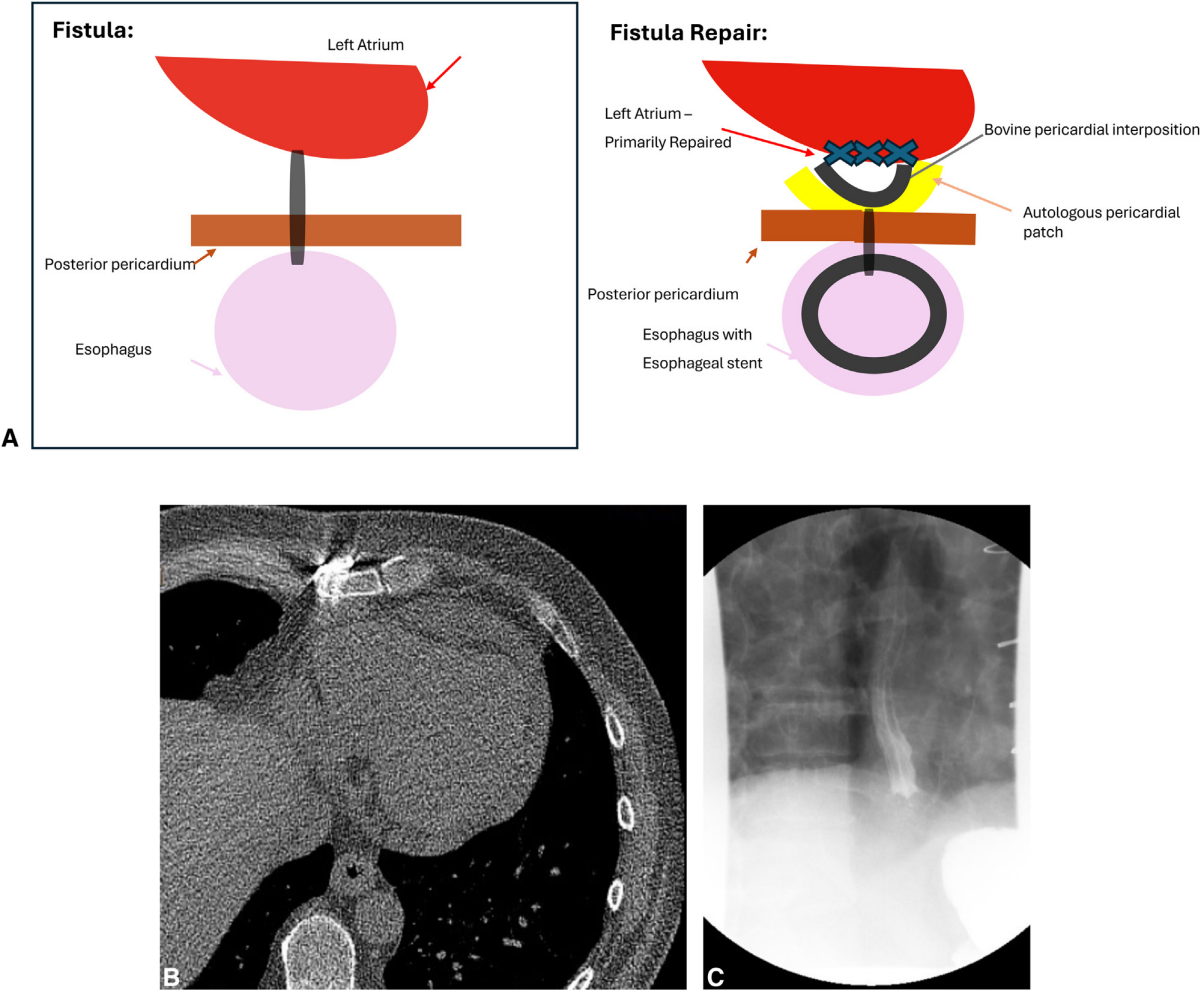
A, Before and after repair. B, Postrepair computed tomography scan. C, Esophagram after stent.

A median sternotomy approach was used to place the patient on cardiopulmonary bypass (ascending aortic and bicaval cannulation). Antegrade Del Nido cardioplegia was used to achieve cardiac arrest. As depicted in Video 1, the LA was accessed via interatrial groove and pulmonary vein cuffs were created, as in lung transplantation. Dense adhesions posterior to the LA were dissected free and devitalized. Left atriotomy was performed and a Cosgrove retractor was introduced to expose the heart. Diameter of the fistulous tract at the posterior wall of the LA was approximately 5 mm and 1 cm at the level of the posterior pericardium (Figure 1, *B*). The posterior pericardium was imbricated over the fistula tract utilizing Vicryl (Ethicon Inc) suture. Pedicled anterior pericardium was mobilized and reinforced pericardial repair with interrupted Vicryl sutures (Figure 2, *A*). Next, a piece of bovine pericardium ∼10 cm in diameter was placed as an interposition patch and sutured with Vicryl. The atriotomy was closed with 4-0 Prolene (Ethicon Inc) in 2 layers and standard decannulation and chest closure were performed with placement of drains posterior to the LA.

Next, attention was turned to the esophagus. Esophagogastroduodenoscopy (EGD) revealed pinpoint mucosal defect at 27 cm from the incisors and covered with a self-expanding esophageal stent (120 × 23 mm). Additionally, a percutaneous gastrostomy tube was placed for enteral feeding.

The postoperative course was uneventful save for minor tube feed intolerance. He was discharged on postoperative day 10 and returned for repeat EGD with stent removal on postoperative day 45 with no further concern for perforation, clinically or on imaging (Figure 2, *B* and *C*).

## Comment

AEF is a rare complication of AF with incidence between 0.03% and 1.5% and mortality between 40% and 80%.^1^ Patients may present with sepsis, hematemesis, neurological symptoms, or chest pain days or weeks after RFCA. CT scan can detect mediastinal air and CT or magnetic resonance imaging of the brain can identify brain lesions.

Isolated esophageal stenting is used for patients with surgical contraindications and is associated with significant morbidity and mortality.^2^, ^3^, ^4^, ^5^ When possible, operative intervention improves survival.^E1^^,^^E2^ The tenets of operative repair of AEF include identification of the fistula, excision and debridement of contaminated tissue, repair of the LA (direct or pericardial patch) and esophageal repair (direct or patch), stent, and rest (ie, nothing by mouth).

Surgical approaches for AEF management include left thoracotomy, right thoracotomy, or sternotomy in combination with a right thoracotomy.^3^^,^^5^^,^E3, E4, E5, E6 Preoperative imaging guides a surgeon regarding extent of esophageal injury and posterior mediastinal involvement. To gain access to the communication between LA and esophageal injury, median sternotomy gives excellent exposure and easily permits cardiopulmonary bypass. A limitation is suitable exposure for managing more extensive esophageal injury. We utilized a sternotomy approach to assess the AEF with subsequent interposition patching and left atrial debridement with primary repair. We proceeded with EGD after LA repair to mitigate risk of cerebral air embolism.^4^ EGD allowed evaluation of the extent and location of injury. With only a small area of injury, we chose to utilize stenting with distal enteral feeding and bowel rest. No further evidence of sepsis was noted throughout their hospital course. With more extensive esophageal injury, options include creation of a proximal spit fistula together with debridement, repair using muscle flap, or primary repair alone. An intercostal muscle flap may serve as a similar buffer instead of a pericardial patch approach. The sternotomy approach makes for challenging direct esophageal repair. Thoracotomy alone—or a staged repair with sternotomy followed by thoracotomy—has been used for direct esophageal repairE3, E4, E5, E6 in patients with more extensive esophageal injury and mediastinal contamination.

Our successful operative approach included esophageal debridement and exclusion, layered closure with buttressed tissue, and fistula tract excision in combination with esophageal stenting.

### Webcast

You can watch a Webcast of this AATS meeting presentation by going to: https://www.aats.org/resources/management-of-atrio-esophageal-8324.

**Figure:**
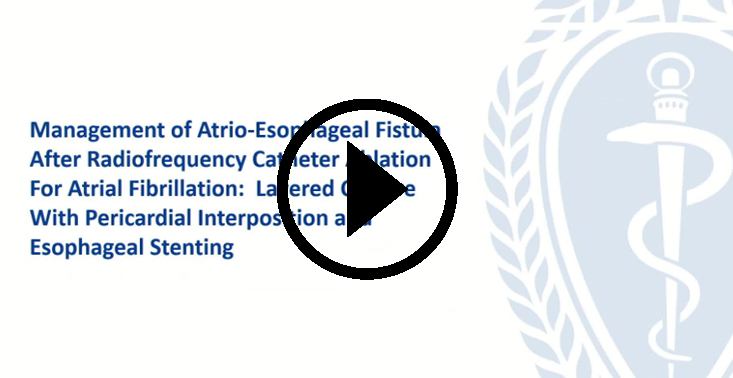

## Conflict of Interest Statement

Dr Mokadam is a consultant for Abbott, Medtronic, J&J, Xylocor, and Carmat. Dr Kneurtz is a speaker and proctor for Intuitive Surgical. Dr Whitson is partially supported through National Institutes of Health National Heart Lung and Blood Institute grant R01HL143000, is on the Clinical Event Committee for Transmedics, and has speaking honoraria from Medtronic. All other authors have no conflicts of interest.

The *Journal* policy requires editors and reviewers to disclose conflicts of interest and to decline handling or reviewing manuscripts for which they may have a conflict of interest. The editors and reviewers of this article have no conflicts of interest.

## Supplementary Data

Video 1 Case video - Management of atrioesophageal fistula. Video available at: https://www.jtcvs.org/article/S2666-2507(24)00356-0/fulltext.
