# Supplementary figures and images for: Management of atrioesophageal fistula after catheter ablation for atrial fibrillation: Layered closure with interposition and esophageal stenting
Source: JTCVS Tech. 2024 Sep 10;28:60–4. doi: 10.1016/j.xjtc.2024.08.023 (PMC11632334; doi:10.1016/j.xjtc.2024.08.023)

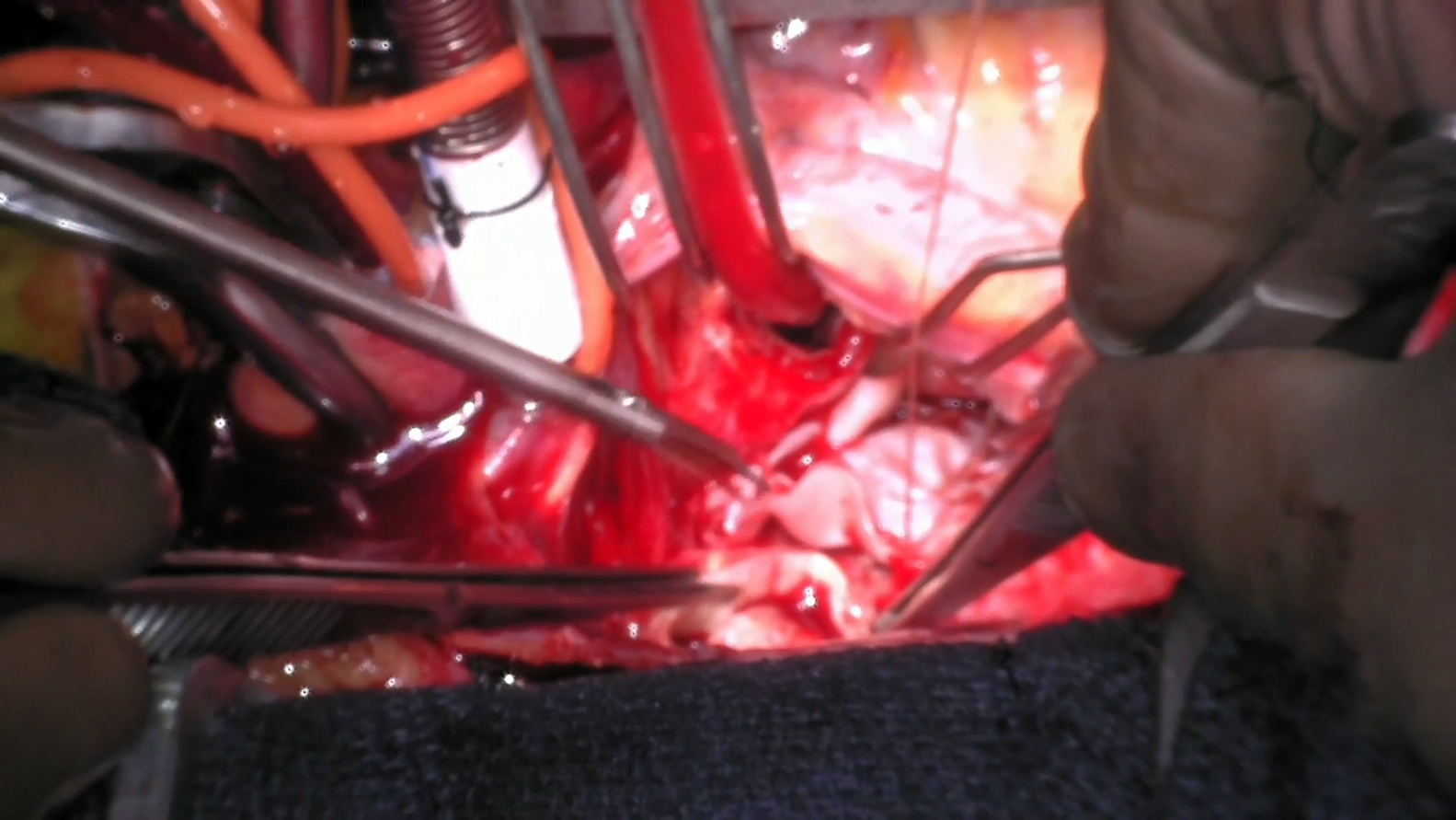

Supplement: Video 1 — Case video - Management of atrioesophageal fistula. Video available at: https://www.jtcvs.org/article/S2666-2507(24)00356-0/fulltext. [file fx3.jpg]
